# Increasing cefazolin use for surgical prophylaxis in penicillin-allergy–labeled patients

**DOI:** 10.1017/ash.2022.360

**Published:** 2023-01-11

**Authors:** Kathryn A. VanderVelde, Sarah L. Suppes, Katherine A. Gibbs, Kevin H. Latz, Angela C. Vanderpool, Rana E. El Feghaly, Jennifer L. Goldman

**Affiliations:** 1 Department of Pediatrics, Children’s Mercy Kansas City, Kansas City, Missouri; 2 University of Missouri-Kansas City, Kansas City, Missouri; 3 Patient Advocate Services, Children’s Mercy Kansas City, Kansas City, Missouri; 4 Department of Orthopedic Surgery, Children’s Mercy Kansas City, Kansas City, Missouri

## Abstract

**Objective:**

Penicillin (PCN) allergy labels affect antimicrobial selection for surgical prophylaxis. We aimed to increase the percentage of cefazolin usage in patients with PCN allergy labels undergoing orthopedic surgery from 50% to 80%.

**Design:**

Quality improvement initiative.

**Setting:**

Children’s Mercy Kansas City (CMKC), a freestanding children’s hospital.

**Patients:**

Children scheduled for an orthopedic surgery (excluding spinal surgery) at CMKC who had a PCN allergy label and received a perioperative antibiotic.

**Methods:**

No standardized process existed to identify and clarify PCN-allergic–labeled patients preoperatively. We developed a process for patient identification combined with a pharmacist phone interview for PCN allergy clarification. In plan–do–study–act (PDSA) part 1, we implemented a computer-generated patient list. In PDSA part 2, we combined automated identification with a phone interview. In PDSA part 3, we enhanced the patient list, making it timely and concise. In PDSA part 4, we included a PCN allergy clarification electronic survey to caregivers via the electronic medical record.

**Results:**

Cefazolin use in PCN-allergic surgical patients increased from 50% to 74% following interventions. Patients who had their PCN allergy label clarified were 4 times more likely to receive cefazolin compared to those whose allergy labels were not clarified (OR, 4.21; 95% CI, 1.68–11.61; *P* = 0.003). Moreover, 90% of patients received cefazolin when their PCN allergy was clarified and cefazolin was recommended. When a PCN allergy label was not clarified, only 59% of patients received cefazolin.

**Conclusions:**

Appropriate clarification and documentation of PCN allergy labels increases the use of cefazolin for surgical prophylaxis.

Antibiotic prophylaxis is recommended for many surgeries, and cefazolin, a first-generation cephalosporin, is often the drug of choice. Cefazolin is deemed preferable to prevent surgical-site infections due to its spectrum of antimicrobial coverage. Cefazolin is also inexpensive and has a short duration of action.^1^ Sometimes, cefazolin is unnecessarily avoided for a patient with a documented penicillin (PCN) allergy. A common myth is that ∼10% of patients with a PCN allergy history will also experience an allergic reaction if administered a cephalosporin. This rate is falsely elevated partially because of contamination of early preparations of cephalosporins with trace amounts of PCN.^2^ More recent observational studies have reported cross-reactivity rates for a severe PCN and cephalosporin allergy to be between 0.17% and 0.7%.^3–5^ These studies have uncovered the association between allergy cross-reactivity and structurally similar side chains rather than the β-lactam ring itself. Thus, cefazolin should be avoided only in patients with severe PCN allergies.

Approximately 5 million children in the United States are labeled PCN allergic.^6^ Only 1%–3% of these patients have a severe PCN allergy and should avoid cefazolin.^4^ Confusion among patients and practitioners about the definition of true allergy and cross reactivity, in addition to inaccurate documentation of drug allergy labels, leads to recommendations for alternative antimicrobial therapy with the potential for lack of efficacy, increased cost, and greater risk of adverse events.^7,8^

At Children’s Mercy Kansas City (CMKC), 6.6% of surgical patients receiving perioperative antibiotics are labeled PCN allergic. Many of these patients receive a cefazolin alternative unnecessarily.^9^ Intraoperative antibiotic institutional guidelines provide cefazolin alternatives in the setting of a drug allergy without context for allergy clarification and interpretation. A process exists to verify drug allergy labels preoperatively, but no process has been developed to fully clarify PCN allergies preoperatively. Verifying a drug allergy involves acknowledgement of an allergy label in the chart, whereas clarifying an allergy involves obtaining specific information regarding the allergy details including timing of a reaction, reaction type and severity, and use of related medications since the event. Allergy clarification often identifies PCN-allergy–labeled patients who likely can safely tolerate cefazolin instead of resorting to an antibiotic alternative. When cefazolin can be used instead of vancomycin, intraoperative time is decreased and the likelihood of surgical-site infections is decreased.^10,11^ Clindamycin, another noncefazolin alternative, has decreasing coverage against *Staphylococcus aureus*.^12^ Prior studies at CMKC reported that without clarification, only 26% of patients with PCN allergy labels received cefazolin for surgical prophylaxis. Therefore, a need was identified to standardize the perioperative antibiotic selection process in PCN-allergic patients undergoing surgery.^9^ Our efforts focused on orthopedics because the surgeons identified the selection of antibiotics in PCN-allergic patients challenging and requested guidance. The primary aim of this quality improvement initiative was to increase cefazolin usage in orthopedic nonspinal surgery patients with a PCN allergy label perioperatively from 50% to 80%.

## Methods

### Population

All patients scheduled at CMKC for nonspinal orthopedic surgery with a PCN-class drug-allergy label who received a perioperative antibiotic were included in this project. PCN-class drug allergy included penicillin, amoxicillin, amoxicillin-clavulanate, ampicillin, ampicillin-sulbactam and piperacillin-tazobactam.

This quality improvement project took place at CMKC, a 314-bed freestanding children’s hospital and its 52-bed satellite location. The antimicrobial stewardship program (ASP) in the division of pediatric infectious diseases, the hospital-wide pharmacovigilance program, and the department of pediatric orthopedic surgery collaborated for this project. CMKC uses electronic medical record (EMR) software (Cerner, Kansas City, MO) for inpatient and outpatient care. The project was considered a quality improvement project not requiring submission to the institutional review board.

### Existing pharmacovigilance program

CMKC has a hospital-wide pharmacovigilance program that focuses on the identification, clarification, and documentation of adverse drug reactions.^13,14^ The pharmacovigilance pharmacist utilizes clinical history and the available medical record to categorize the patient’s PCN reaction as allergic (ie, immune mediated) or nonallergic (eg, drug side effect). Severity of the reaction is also categorized including mild severity in which the drug was continued, moderate severity in which the implicated drug was discontinued and/or the reaction required treatment but was not life-threatening, or a severe reaction that was life-threatening and/or required hospitalization and/or delayed hospital discharge.

### Planning the interventions

This multidisciplinary quality improvement team was led by 1 pediatric infectious diseases fellow and 1 clinical pharmacovigilance pharmacist. The team also included 3 operating-room pharmacists, 1 infectious disease attending physician, 1 quality improvement hospital expert, 1 immunology attending physician, 14 orthopedic surgeons, and 5 orthopedic nurse practitioners. We developed a process map of the path preceding orthopedic surgery to identify opportunities for PCN-allergy label clarification in the workﬂow. A patient engages with the healthcare system several times from the point of presurgical evaluation to the operating room, and drug allergies are frequently verified, but not clarified, during these interactions. The process map revealed that although PCN-allergy label verification occurred at multiple time points, there was no standardized or dedicated time for PCN-allergy label clarification in the workﬂow.

A key driver diagram helped reveal that identification of drug allergy labels by medical providers, adequate documentation of allergy labels, drug selection clarification and justification, and communication amongst medical team members and the family regarding medication recommendations were the primary drivers in achieving increased cefazolin usage. Using a PICK (possible, implement, challenge, kill) chart developed by our team to compare impact with effort, we hypothesized that reviewing patients scheduled for orthopedic surgery would likely provide the highest impact without requiring additional resources.

### Interventions

We used plan–do–study–act (PDSA) cycles from March 2019 to December 2020 to identify opportunities for PCN allergy clarification (Table 1). Prior to the initiation of this project, any member of the surgical team could refer patients to a pharmacovigilance pharmacist for drug-allergy label review and parental phone interview for allergy clarification prior to surgery.

**Table 1.** Summary of Interventions

Cycle	Intervention	Interval
Baseline	Physician-based referral with phone drug-allergy clarification interview	August 2018–January 2019
PDSA 1	Developed a computer-generated patient identification tool to obtain a list of all PCN-allergy–labeled patients and provided education	March 2019–May 2019
PDSA 2	Tool from PDSA part 1 filtered for specific criteria combined with phone interviews by the quality improvement team	June 2019–Sept 2019
PDSA 3	Process from PDSA part 2 continued but included all patients scheduled for nonspinal surgery and retimed to align with weekly huddle	Oct 2019–April 2020
PDSA 4	Addition of patient portal survey to the PDSA 3 process	Aug 2020–Dec 2020

Note. PDSA, plan–do–study–act; PCN, penicillin.

In PDSA 1, a computer-generated patient identification tool was manually filtered by the quality improvement team to include the defined population of patients who were anticipated to need antimicrobial prophylaxis for their orthopedic surgery. In addition, the quality improvement team provided education to the schedulers, nurses, and providers involved with orthopedic procedures on how to identify PCN-allergic patients.

For PDSA 2, the patient’s caregivers were contacted by phone prior to surgery and interviewed regarding the details of the patient’s drug-allergy label (Supplementary Table 1). Additional information was also acquired by contacting the patient’s primary care physician and preferred pharmacy to determine whether the patient had received and tolerated PCN despite the allergy label. Data obtained from these interviews were communicated via the EMR messaging system to the surgical team and operating room pharmacists (Supplementary Table 2).

**Table 2.** Surgical Patients With a PCN Allergy Label Who Underwent Label Clarification, and the Odds of the Allergy Label Being Clarified

Cycle	Weeks, No.	Surgical Patients with PCN Allergy Label Eligible for Clarification, No.	Patients With PCN Allergy Label Clarified, %	Odds Ratio	*P* Value	95% Confidence Interval
Baseline	26	90	4.44	Reference	…	…
PDSA 1	8	16	25.00	7.17	.0107	1.52–34.20
PDSA 2	15	38	63.16	36.86	<.001	12.18–140.69
PDSA 3	20	46	69.57	49.14	<.001	16.60–185.25
PDSA 4	19	39	51.28	22.63	<.001	7.58–84.93

Note. PCN, penicillin; PDSA, plan–do–study–act.

PDSA 3 included a review of all patients scheduled for orthopedic surgery, regardless of whether antimicrobial prophylaxis was anticipated for surgery.

PDSA 4 involved distributing an electronic allergy interview that was shared with families through the EMR patient portal (Supplementary Table 1). This approach was intended to replace the need for a phone interview to gather information about the PCN allergy and to make the process more efficient. All patients received follow-up phone calls to confirm information reported on the collection tool or, if the tool was not completed, to report PCN allergy clarification.

### Measures

The main outcome measure was the percentage of patients with a PCN allergy label who underwent a nonspinal orthopedic surgery and received cefazolin for surgical prophylaxis per week. Process measures included the number of patients receiving perioperative antibiotics with PCN allergy labels undergoing nonspinal orthopedic surgery for whom their drug-allergy label could be clarified, the rates of PCN-allergy clarification, and cefazolin administration. Our balancing measure was adverse drug reactions associated with receiving cefazolin as a perioperative antibiotic. To identify possible cases in which a patient had a reaction to an antibiotic in the peri- and postoperative periods, the anesthesia record and postoperative notes were reviewed to identify any events during and after the procedure.

### Analysis

Outcome and process measures were assessed on a control chart and using Microsoft Excel (Microsoft, Redmond, WA) and QI Macros 2022. Shewhart control chart rules were used to shift the central line and control limits.^15^ A shift was defined as 8 or more points in a row above or below the central line and a trend was defined as 6 or more points in a row increase or decreasing. The odds ratio that the PCN allergy was clarified in each PDSA cycle, relative to the baseline period, was calculated using an unadjusted logistic regression model. Similarly, a logistic regression model was used to compare the odds of receiving cefazolin based on whether the PCN allergy was clarified during the PDSA cycles.

## Results

The computer-generated patient identification tool implemented in PDSA part 1 identified 16 patients with PCN allergy labels scheduled for orthopedic surgery. Among these patients, 4 had their PCN allergy clarified, and cefazolin was recommended in all 4 cases. Subsequently, these 4 patients safely received and tolerated cefazolin. Of the 12 patients who did not have their PCN allergy clarified, only 7 (58%) received cefazolin.

In PDSA part 2, 38 patients were identified in 15 weeks (2.5 patients per week). The combination of the computer-generated tool for patient identification with the phone interview resulted in clarification of 24 PCN allergy labels (63%). Two patients had phone interviews that revealed a concern for a severe PCN allergy. For these patients, a cefazolin alternative was recommended for surgical prophylaxis if the patient was unable to undergo PCN allergy testing prior to surgery. Of the 22 patients who were recommended to receive cefazolin for surgical prophylaxis, 18 (82%) received cefazolin and all tolerated the antibiotic. During PDSA cycle 2, the percentage of those whose label was clarified to increased 63% from 25% in PDSA 1.

In PDSA part 3, we identified 46 PCN-allergy–labeled patients who underwent surgery and received antibiotic prophylaxis in 20 weeks (2.3 patients per week). Also, 32 patients had their PCN-allergy labels clarified, and cefazolin was recommended in 26 of these cases. Among these 26 patients, 25 were prescribed cefazolin, and all tolerated the antibiotic. In PDSA 3, we identified 5 patients interested in PCN drug-allergy testing. Of the patients who underwent testing, 100% had their PCN allergy label removed.

In PDSA part 4, we implemented an electronic allergy data collection tool that further automate the patient interview process (Supplementary Table 1). Moreover, 13% of surveys distributed were returned, and 50% of returned surveys required phone interviews for additional clarification.

**Fig. 1.**
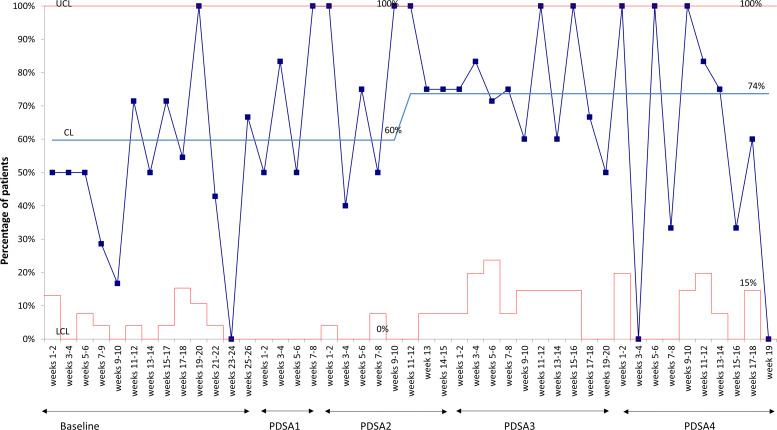
Percentage of patients with a PCN-allergy label undergoing surgery who received cefazolin.

For the outcome measure, the overall percentage of surgical patients with a PCN allergy label who received cefazolin increased from 50% at baseline to 74% during the study period (Fig. 1). For process measures, the odds of a PCN allergy label being clarified significantly increased during the PDSA cycles compared to the baseline (Table 2 and Supplementary Fig. 1). At baseline, <5% of patients with a PCN allergy label were clarified. In PDSA 1, those whose PCN allergy labels were clarified increased to 25%, and in PDSA parts 2–4, >50% of PCN allergy labels were clarified. When evaluating cefazolin prescribing, patients who had their PCN allergy label clarified were 4 times more likely to receive cefazolin compared to those whose PCN allergy labels were not clarified (OR, 4.21; 95% CI, 1.68–11.61; *P* = .003). No adverse reactions to antibiotics were identified in the perioperative or postoperative period.

Based on the background knowledge that ∼6.6% of surgical patients at CMKC have a PCN allergy label^9^ and ∼52 surgeries are performed by orthopedic surgeons per week (80% of which are nonspinal), our anticipated PCN-labeled patients requiring clarification would be 2.7 patients per week. However, requests for allergy clarification were much lower than expected at the beginning of this project. From August 2018 through January 2019, 90 nonspinal surgical orthopedic patients were labeled as PCN allergic in the chart; however, only 4 of these patients were referred for evaluation by the orthopedic surgery team. Of the 4 patients referred, cefazolin was recommended for 3, and all 3 safely received cefazolin. For the fourth patient, the recommendation was to avoid cefazolin and the patient safely received a cefazolin alternative. Of the 86 patients not referred, 46 (53%) received cefazolin perioperatively despite the PCN allergy label and 40 received a cefazolin alternative.

## Discussion

A PCN-allergy label is often lifelong because few patients are rechallenged to assess tolerability, resulting in more expensive, less effective, and broader-spectrum antibiotics being used for surgical prophylaxis.^10^ Vague, incomplete, or inaccurate drug-allergy histories can negatively affect patient safety and patient care, and can disrupt workflow for clinicians.^16^ Although previous studies have clarified patient allergy labels, few studies have used a standardized drug-allergy interview process to clarify drug allergies prior to surgery. Through use of a standardized review process, allergy clarification prior to surgery can increase PCN label clarification from 4.4% at baseline to >50% of all patients undergoing orthopedic surgery. Overall, 90% of patients received cefazolin when their PCN allergy label was clarified, and cefazolin was recommended. When a PCN allergy label was not clarified, only 59% of patients received cefazolin.

The systems put in place during this quality improvement initiative continue through our hospital-wide pharmacovigilance program led by a pharmacist. However, the amount of time spent on this project weekly was not studied. Many variables must be considered when attempting to estimate workload: number of patients with a documented allergy, number of patients scheduled per week, and type of surgery. This research requires dedicated resources to perform the allergy reviews and provide antibiotic guidance to the surgical team. Expanding to additional surgical subspecialties requires the investment and interest from the surgical teams and common use of perioperative antibiotics. To date, we have not expanded beyond orthopedics.

Drug allergy clarification prior to surgery is feasible and effective, and it has the potential to alter a patient’s future antimicrobial selection toward the use of a narrower, more effective drug choice. The presurgical evaluation poses an opportunity for a drug-allergy clarification interview by a trained professional to improve allergy documentation and increase use of appropriate antibiotic selection for perioperative antimicrobials.

This study had several limitations. Adoptive or foster parents were unable to provide medical histories, and we also encountered unreturned phone calls and unavailable caregivers during interviews. We observed a high baseline use of cefazolin in patients with PCN allergy labels undergoing orthopedic surgery. The reasons why cefazolin was prescribed in PCN- allergy–labeled patients who had not undergone clarification was not investigated, but it may be due to heightened awareness of orthopedic surgeons involved in this study. The electronic allergy data collection tool used in PDSA cycle 4 had limited success, and further evaluation of the effectiveness of this tool was not conducted. Additionally, surgical site infections were not evaluated as an outcome. Factors that may have limited internal validity included counting patients who were unable to be contacted as unclarified, inclusion of patients who were scheduled for surgery on the day of list generation, and a small number of providers tasked with clarifying PCN allergy labels.

Further collaborative efforts with surgical specialties are needed to evaluate patients labeled with allergies to antibiotics. From our study, the acute impact of the benefit of allergy clarification prior to orthopedic surgery is clear, and this clarification will likely have a lifelong impact on a patient’s antimicrobial selections.

## Supplementary material

For supplementary material accompanying this paper visit https://doi.org/10.1017/ash.2022.360. click here to view supplementary material
