# Supplementary material for: Increasing cefazolin use for surgical prophylaxis in penicillin-allergy–labeled patients
Source: Antimicrob Steward Healthc Epidemiol. 2023 Jan 11;3(1):e11. doi: 10.1017/ash.2022.360 (PMC9879898; doi:10.1017/ash.2022.360)
Supplement: Supplementary file 1 [file ashsup.zip › S2732494X22003606sup001.docx]

**Supplemental Table 1. Standardized Penicillin Allergy Review**

| 1. Antibiotic (penicillin, amoxicillin, amoxicillin-clavulanate, Augmentin, etc.): |
| --- |
| 2. Age of child at time of reaction: |
| 3. What was the antibiotic prescribed for? (ex, strep throat, ear infection, etc.) |
| 4. Describe the reaction your child had: |
| 5. Select any specific signs and symptoms (place an X in front of underscore):  (X_ example)  _ anaphylaxis  _ shortness of breath  _ hives (If so, where: _)  _ angioedema (tongue/lip/facial swelling)  _ throat closing  _ itching (If so, where: _)  _ rash (If so, where: _)  _ dizziness  _ nausea  _ vomiting  _ swelling (If so, where: _)  _ stomach upset/pain  _ diarrhea  _ dizziness  _ drowsiness  _ weakness  _ muscle pain/soreness  _other: (please list) |
| 6. How soon after starting the medication did the reaction happen? (please write number)  (12_example)  _number of doses  _hours  _days  _weeks  _other |
| 7. Did you seek medical attention for the reaction?  _Y  _N  If yes, were you seen in the:  _ Emergency Department/Urgent Care  _ Doctor’s office  _ Admitted to the Hospital  _Other  _n/a |
| 8. Was the medication stopped by a doctor?  _Y  _N |
| 9. Did your child require medical treatment for the reaction?  _Y  _N  If yes, what medications were given (ex: Benadryl, diphenhydramine, epinephrine, steroids): _ |
| 10. What happened after the antibiotic was stopped: _ |
| 11. Are there any antibiotics your child has taken and tolerated (ex: amoxicillin (Amoxil®), amoxicillin/clavulanate (Augmentin®), cephalexin (Keflex®), cefdinir (Omnicef®), cefixime (Suprax®)? _ |
| 12. Has your child had drug allergy testing?  _Y  _N  If yes, what were the results |
