# Supplementary material for: Increasing cefazolin use for surgical prophylaxis in penicillin-allergy–labeled patients
Source: Antimicrob Steward Healthc Epidemiol. 2023 Jan 11;3(1):e11. doi: 10.1017/ash.2022.360 (PMC9879898; doi:10.1017/ash.2022.360)
Supplement: Supplementary file 1 [file ashsup.zip › S2732494X22003606sup003.docx]

**Supplemental Table 2. Example messaging sent to surgical team, operating room pharmacists, and patient electronic medical record portal**

| **Example 1:** After reviewing patient X’s reported amoxicillin allergy, we have been able to clarify that he has history of a **moderate penicillin allergy**. His reaction was pruritic rash when he was an infant after a week of taking amoxicillin.  If antibiotic prophylaxis for his upcoming orthopedic procedure is warranted, then **cefazolin would be recommended**.  Penicillin-allergy testing is also recommended for future use of penicillins.  *Brief history:* Per mom - Patient was prescribed amoxicillin 10 years ago when he was an infant. He then experienced a red, itchy rash a week after taking the antibiotic. Amoxicillin was stopped. Mom was unable to recall any other specific details. Mom did state he has since received and tolerated a "similar medication" and azithromycin. Pharmacy records show he was prescribed cephalexin in Sept 2016 and azithromycin in Oct 2019. |
| --- |
| **Example 2:** After reviewing patient Y’s reported penicillin and amoxicillin/clavulanate allergies, we have been able to clarify that he has a **severe penicillin allergy** as we have no record of him receiving a penicillin-derivative since his reported allergic reaction.  If antibiotic prophylaxis for his upcoming orthopedic procedure is warranted, then **an alterative to cefazolin should be considered** and we would suggest referral for penicillin allergy testing. Previous surgery in July 2018 he received clindamycin.  *Brief history:*  Amoxicillin/clavulanate reaction: Per mom - Patient was prescribed amoxicillin/clavulanate years ago when he was much younger. After taking, he began vomiting and was unable to keep anything down. PCP was contacted and amoxicillin/clavulanate was discontinued.  Penicillin reaction: Per mom - Patient was prescribed penicillin a couple years ago for either otitis media or a sore throat. After taking 1-2 doses he began to experience an itchy "severe" rash and difficulty breathing. He was taken to an outside ED and was given Benadryl and a shot of something. He was monitored then sent home. Mom is unable to recall other antibiotics that patient has since tolerated. Pharmacy records indicate that he has been prescribed cefuroxime in Feb 2020 and cefdinir in Nov 2018. |
| **Example 3:** After reviewing patient Z’s reported penicillin allergy, we have been able to clarify that he has **does not have an allergy to penicillin**. He has taken and tolerated amoxicillin in the past with no reaction. He has also received cefazolin in the past with no documented reaction.  If antibiotic prophylaxis for his upcoming orthopedic procedure is warranted, then **cefazolin would be recommended**.  *Brief history:* Per mom - Patient has taken and tolerated amoxicillin. Mom would like for providers to be aware that patient Z’s grandpa experiences a severe reaction to penicillin. Mom also experiences a rash herself with penicillin. Patient Z has never experienced an adverse reaction to any medication. Patient Z has also received and tolerated cefazolin in the past per hospital records on June 1, 2018. |
