# Supplementary figures and images for: Increasing cefazolin use for surgical prophylaxis in penicillin-allergy–labeled patients
Source: Antimicrob Steward Healthc Epidemiol. 2023 Jan 11;3(1):e11. doi: 10.1017/ash.2022.360 (PMC9879898; doi:10.1017/ash.2022.360)

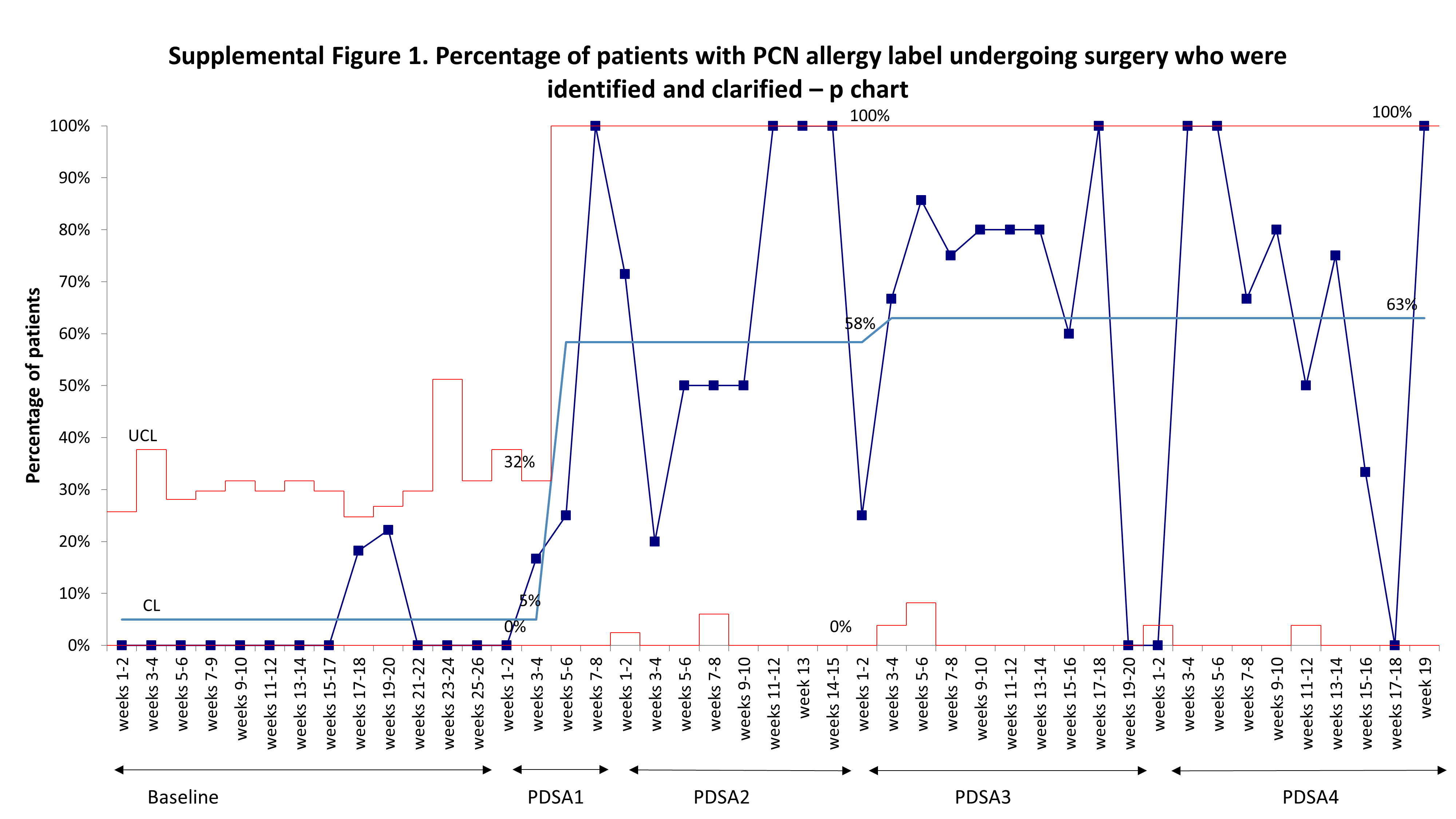

Supplement: Supplementary file 1 [file ashsup.zip › S2732494X22003606sup002.tif]
